# Retrospective evaluation of prehospital triage, presentation, interventions and outcome in paediatric drowning managed by a physician staffed helicopter emergency medical service

**DOI:** 10.1186/s13049-015-0177-0

**Published:** 2015-11-06

**Authors:** Alan A. Garner, Claire L. Barker, Andrew D. Weatherall

**Affiliations:** CareFlight, 4 Barden Street, Northmead, NSW 2152 Sydney, Australia

**Keywords:** Drowning, Immersion, Paediatric, Prehospital, Resuscitation

## Abstract

**Background:**

Drowning patients may benefit from the advanced airway management capabilities that can be provided by physician staffed helicopter emergency medical services. The aim of this study is to describe paediatric drowning patients treated by such a service examining tasking systems, initial physiology at the incident scene, survival and neurological outcome.

**Methods:**

Retrospective analysis of paediatric drowning victims over a 5- year period. Case identification system, patient age, site of drowning, presence or absence of cardiac output, first Glasgow Coma Scale (GCS) score and interventions were collected from prehospital notes, and survival and neurological outcomes from hospital and rehabilitation notes.

**Results:**

The P-HEMS direct case identification system operating in parallel with a central control system identified all severe drowning cases but 3 of 7 cases (43 %) were missed when the central control system operated in isolation. All severe drowning cases (22) identified for P-HEMS response were intubated and transported directly to a paediatric specialist centre. Intubation required adjuvant anaesthesia in 10 (45 %) cases. All children with GCS greater than eight on arrival of the P-HEMS survived neurologically intact. Seven of eight children with a GCS between four and seven survived without neurological impairment and all children with a GCS greater than three survived. Four of twelve asystolic children survived including one child who at 18 months post drowning is neurologically normal. All children who survived had return of spontaneous circulation prior to arrival in the emergency department.

**Conclusions:**

P-HEMS played a significant role in the management of severe paediatric drowning in this case series. Requirement for P-HEMS only interventions were high and all identified cases were transferred directly to a paediatric specialist centre. Discontinuation of the P-HEMS direct case identication system that operated during the majority of the study period resulted in deterioration in system performance with some paediatric drowning cases subsequently not identified for P-HEMS response being transported to adult hospitals.

## Text

### Background

Drowning claims the lives of approximately 300 people per year in Australia [1] and was responsible for 7 % of deaths in Australian children in 2004–5 [2]. It is the leading cause of unintentional injury death in young children worldwide [3].

There is robust Australian information about the patient demographics and site of fatal drownings [1, 4] but little information on the complete [fatal and non-fatal] prehospital population and their outcomes in terms of mortality, treatment and longer term morbidity. Additionally there is little published data on the involvement of physician staffed helicopter emergency medical services (P-HEMS) in the prehospital management of drowning, intervention rates and the possible effects on outcome. Drowning cases may particularly benefit from the higher level airway management skills that a P-HEMS team brings to the scene. The aim of this study was to describe all paediatric drowning patients treated by a P-HEMS comparing the initial presentation on arrival of the service at the incident scene, interventions performed and survival rates with neurological outcome.

### Methods

Retrospective cohort analysis of patients under the age of 16 years treated for drowning during a 5-year period between April 2007 and April 2012 by the CareFlight P-HEMS service. The definition of drowning used in this study is that accepted at the 2002 World Congress on Drowning [5] and in 2005 by the World Health Organization and includes fatal and non- fatal cases. Drowning is the process of experiencing respiratory impairment from submersion/immersion in liquid. Ethical approval was obtained from the Sydney Children’s Hospital Network Human Research Ethics Committee.

The CareFlight P-HEMS service covers a population of approximately 4.5million people in a predominantly suburban context in the greater Sydney area with a radius of operation of 100 km from their base, which is located at Westmead near to the demographic centre of the city. The case identification and dispatch system for the service has been described previously [6]. P-HEMS teams were tasked as part of a comprehensive EMS response to patients where the emergency call indicated immersion mechanism and either a reduced level of consciousness or CPR in progress. From 2007 to March 2011 cases were identified either by P-HEMS crew directly screening the NSW Ambulance computer assisted dispatch (CAD) system or by a dedicated paramedic in a central control room as previously described [6]. After March 2011 the dispatch system reverted to the centralized dispatch system only as NSW Ambulance withdrew access for the P-HEMS crew to the CAD system.

Incident scene data collected followed the Utstein template for reporting of drowning outcomes [7] including age, place of drowning, provision of bystander cardiopulmonary resuscitation (CPR), presence or absence of cardiac output and first Glasgow coma score (GCS) recorded by the P-HEMS service, and interventions required at the incident scene and in the emergency department. Neurological outcomes including physician neurological assessment at discharge and subsequent neurology or rehabilitation specialist assessments were collected. Neurological impairment as judged and recorded by the treating hospital clinician in the medical record at last recorded contact either at discharge or subsequent follow up was coded according to the Paediatric Cerebral Performance Category (PCPC) [8]. Results of respiratory and/or cerebral imaging were collected. Data sources were paramedic, P-HEMS and hospital medical notes.

## Results

It is known from the previous dispatch study [6] that all paediatric drowning cases with an Injury Severity Score (ISS) > 15 recorded in the NSW Trauma Registry occurring during the operational hours of the HEMS service were identified by the parallel tasking system that operated till March 2011. All such cases were intubated by the P-HEMS team and transported directly to a paediatric specialist centre. In the one year period after March 2011 when only the centralised dispatch system was in operation there were seven drowning cases with ISS >15 during the HEMS operational hours, of which only four (57 %) were identified for P-HEMS response. Two of the remaining three cases were transported by road paramedics to adult trauma centres then secondarily transported to a paediatric specialist centre where they subsequently died, arriving at the specialist centre more than four hours after the incident in both cases. As these three missed cases were not treated by the reporting P-HEMS service they are not included in the present study. Additionally one of the four patients identified for P-HEMS response was managed by a NSW Ambulance physician team and is also not reported.

Therefore included in this report are the 42 paediatric patients transported by the reporting P-HEMS service with immersion injury during the five year study period. The median age of the children was 2.8 years (range 0.6–12.2). More patients were male (*n* = 26) than female (*n* = 16). Two thirds of children were less than 4 years of age. Twenty nine had an ISS >15 (altered level of consciousness or documented cardiac arrest) and 13 had minor immersion injury. Place of immersion is shown in Table 1. Table 2 shows interventions performed prior to hospital arrival. Subsequently eleven patients were transported by helicopter, 24 were transported by the P-HEMS team via road ambulance to a paediatric specialist centre and seven cases were transported by road paramedics alone after assessment by the P-HEMS team as they were physiologically stable with normal respiratory examinations.

**Table 1:** Immersion incidents according to place of accident. Other includes ocean, lagoon, river and dam

Place of immersion	Number	Median Age [range]
years		
Pool	31	2.8 (1.2–12.6)
Bathtub	5	1.0 (0.6–7.7)
Other	6	4.2 (1.9–5.9)

**Table 2:** Interventions prior to hospital arrival

Intervention	Number
Bystander CPR	28
Intubation	22
Crystalloid bolus	16
Intraosseous access	17
CPR by P-HEMS	13
Induction of anaesthesia by P-HEMS	10

### Survival

Ten children died within 30 days of the incident, these children ranged from 1.1 to 7.7 years of age. Five were declared dead in the emergency department and five died between two and nine days later. All children who died had a GCS of 3 and were in cardiorespiratory arrest at the accident scene. One child died at 17 months post immersion from complications arising from the injury so is therefore considered an immersion death as per the Utstein reporting template [7].

Four children with GCS 3 survived to at least six months after the accident, although as mentioned above one of these died at 17 months post immersion. Three of these children were in cardiopulmonary arrest when the P-HEMS team arrived. All children who survived had a spontaneous circulation on arrival in the emergency department. Table 3 shows survival and neurological outcome by GCS. Twelve out of 17 ventilated children who were admitted to the paediatric intensive care unit (PICU) survived.

**Table 3:** Outcomes by GCS at initial assessment. PCPC, Paediatric Cerebral Performance Category

	GCS 15-13	GCS 12-8	GCS 7-4	GCS 3
Normal (PCPC = 1) or return to baseline	16	4	7	1
Neurological impairment	0	0	1	2
(PCPC = 2–5)				
Died (PCPC = 6)	0	0	0	11

### Neurological outcome

No child with a GCS greater or equal to eight on arrival of the P-HEMS had neurological impairment at the time of follow up. Outcomes of all children with GCS less than eight are shown in Table 4. All twenty two children with GCS below eight were intubated and ventilated at the accident scene by the P-HEMS team, ten (45 %) requiring adjuvant anaesthesia. Of four children who survived with neurological impairment, three had a GCS of three on arrival of the P-HEMS team. One child however, who suffered an asystolic cardiac arrest, survived and had a normal neurological examination as assessed by a paediatric rehabilitation specialist 18 months after the incident (PCPC =1).

**Table 4:** Neurological outcome of children with an initial GCS < 8

Age	Asystole first reported rhythm	Initial GCS	Cardiac output on ED arrival	Outcome	PCPC score	Outcome Comments
5.3	Y	3	N	Died ED	6	
1.1	Y	3	N	Died ED	6	
1.5	Y	3	N	Died ED	6	
3.5	Y	3	N	Died ED	6	
2.0	Y	3	N	Died ED	6	
2.5	Y	3	N	Died day 4	6	MRI- multiple areas of infarction involving brain stem, deep nuclei and cortex
2.8	Y	3	Y	Died day 2	6	Bradycardic on ED arrival, MRI- diffusely swollen brain, transtentorial and foramen magnum herniation, no arterial or venous flow
6.4	Y	3	Y	Died day 2	6	MRI extensive cerebral and cerebellar oedema with BG and brainstem involvement. Tonsillar herniation. No flow on MRA
7.7	Y	3	Y	Died day 2	6	CT complete loss grey- white matter differentiation, herniation of cerebellar tonsils
1.2	Y	3	Y	Died day 9	6	MR bilateral globus pallidus, hippocampal and bilateral cortical areas of ischaemic injury. No purposeful movements, occasional breaths, intensive care withdrawn.
3.5	Y	3	Y	Died 17 months	6	At 3 months - severe spastic quadriplegia with bulbar palsy, incomprehensible sounds. Died at 17 months
1.3	Y	3	Y	Neurological impairment	4	At 6 months - significant dystonia, trunk hypotonia, epilepsy
1.0	Y	3	Y	Normal	1	CT - early cerebral oedema, MRI no diffusion restriction. Early seizures on PICU. At 18 months -normal neurological and developmental exam
1.7	N	3	Y	Neurological impairment	3	Pre accident diagnosed Aspergers and ADHD. At 1 month – right sided weakness, loss of language. At 3 ½ years - distractible, special help at school
2.2	N	5	Y	Normal	1	At 12 months -ahead in development
2.8	N	5	Y	Normal	1	At 11 months -Normal function
1.2	N	5	Y	Normal	1	Tonic clonic seizures in PICU. At 1 yr 5 months development normal.
2.1	N	6	Y	Normal	1	No neurological deficit at discharge
3.9	N	6	Y	Normal	1	At 7 months normal behavior and skills
9.9	N	6	Y	Baseline*		*Had seizure disorder, autism, ADHD, moderate –severe disability before accident. Returned to baseline state.
1.3	N	7	Y	Neurological impairment	3	At 2 yrs - behaviour problems, aggression, repetitive movements/routines
1.9	N	7	Y	Normal	1	No neurological deficit at discharge

*Patient not neurologically normal at follow up but had returned to pre-incident baseline

**Fig. 1:**
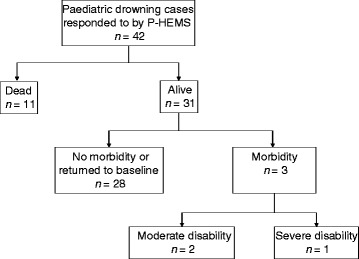
Outcome of the all paediatric drowning cases treated by the P-HEMS service according to the Utstein reporting template

Survival and disability rates are summarized in Fig. 1 according to the Utstein reporting template.

### Respiratory outcome

Thirteen of the twenty non-intubated children had chest x-rays performed in the emergency department and in nine cases changes consistent with aspiration were reported. Four non-intubated children required supplementary oxygen on day one. No non-intubated child developed respiratory symptoms requiring higher respiratory support.

## Discussion

There is little previously published data on the role of P-HEMS in paediatric drowning cases and their utility in this context has yet to be established. There was a very high intervention rate in severely injured (ISS > 15) drowning cases in this series treated by the P-HEMS. All of these patients were intubated with 45 % requiring adjuvant anaesthesia. Consensus guidelines on management of drowning [5] emphasise early restoration of ventilation and circulation. Activation of teams able to provide anaesthesia assisted intubation and direct transport to specialist paediatric centres is therefore consistent with current best practice. P-HEMS are ideally constituted to deliver both advanced airway interventions and direct transport [6]. Definitive evidence that these interventions improve outcome is however not yet available. A large multicenter trial would be required as individual EMS systems are highly unlikely to encounter adequate patient numbers to make such a study feasible.

The previous study of the paediatric trauma case identification system used in Sydney indicated that direct identification by the P-HEMS service significantly outperformed paramedic identification from a central control room, and had profound effects on the overall performance of the Sydney prehospital trauma system [6]. Similarly whilst the P-HEMS case identification system was in operation in this study, all paediatric drowning cases with an ISS > 15 were identified for P-HEMS response and were transported directly to a paediatric specialty centre. After the NSW Ambulance withdrew access by the P-HEMS to the CAD system only four of seven cases were identified. Two of the three non-identified cases were transported by road paramedics to adult trauma centres with delays of several hours before transfer to a paediatric specialist centre. This indicates that further investigation of the effects of cessation of the P-HEMS case identification system should be undertaken and reinstatement of the parallel identification model may be warranted. Beyond the case identification process the system worked well, with all severe drowning cases identified for P-HEMS response receiving intubation and direct transport to a paediatric specialist centre.

The P-HEMS was also very accurate in identifying high risk patients for respiratory complications in this small series. No child that was not intubated during the prehospital phase of care subsequently required intubation in the hospital.

All children with a GCS over 3 on arrival of the P-HEMS survived. Twenty five percent of children who suffered a cardiac arrest survived which is a relatively high survival rate for out of hospital cardiac arrest. For all causes of reported paediatric out of hospital cardiac arrest survival rates range from 6.4 % to 12 % [9–12] with neurologically intact survival in half of these patients [9, 12, 13]. Arrested drowning victims are reported to have better survival rates than the all-cause arrest group. In a pooled review of 41 studies drowning associated arrests had a 22.7 % survival [9]. This is similar to the current series and emphasises the need to aggressively resuscitate paediatric drowning victims who have suffered cardiorespiratory arrest in the pre hospital setting as individual outcomes cannot be predicted.

No child survived who did not have a return of spontaneous circulation before admission to the emergency department. In all causes of paediatric cardiac arrest in a series of over 200 children in Melbourne, Australia less than 1 % survived if spontaneous circulation remained absent on arrival at hospital [10]. In a large US study of 599 out of hospital paediatric cardiac arrests the longest duration of CPR in a survivor with a good neurological outcome was 42 min [14] and a series of children who drowned reports no children receiving more than 25 min CPR having a good outcome [15]. A recent nationwide study of paediatric drowning outcome in the Netherlands found that no child who received CPR for more than 30mins without return of spontaneous survived with a good neurological outcome [16]. Prehospital management is therefore critical and again would suggest a role for P-HEMS in this population.

The decision surrounding cessation of cardiopulmonary resuscitation after drowning has been complicated by case reports of survival after long periods of both submersion [15] and CPR [17, 18, 19] in hypothermic patients. It is likely that hypothermia is only protective if it occurs before irreversible hypoxic-ischaemic cerebral injury which is less likely to occur in Australian water conditions compared to cooler areas of Europe or North America.

The rates of bystander CPR in this series were high, suggesting community education programs are having effect. All but one child with a GCS less than 8 on arrival of the P-HEMS had received bystander CPR, and in all asystolic children CPR was underway. Bystander CPR is associated with improved neurological outcome in children admitted to ED after drowning [20]. In cases of cardiopulmonary arrest secondary to drowning, positive pressure ventilation via mouth or mask is required in addition to chest compressions for resuscitation to be effective. In all causes of paediatric out of hospital arrest rates of favorable outcome increase if breaths are added [13].

An initial GCS above 8 at the scene was associated with full recovery. However a GCS below 8 did not predict a poor outcome, seven out of eight children with initial GCS between four and seven had good neurological outcomes. This is in accordance with other studies which also show initial neurological parameters such as low GCS, lack of response to pain and lack of pupillary reaction do not necessarily predict poor neurological outcomes [21, 22].

One child in this series survived neurologically intact after a period of asystolic cardiopulmonary arrest. Normal survival after cardiopulmonary arrest due to drowning has been well described [23] and in a meta-analysis of 442 drowning associated arrests the intact survival rate was 6 %, putting it well above rates for all other causes of cardiopulmonary arrest [9].

### Limitations of this study

The neurological outcomes reported are short term. None of the children in this series who had a GCS between three and eight and were classified as normal were followed for more than 18 months and none had started school. Long-term cognitive sequelae may come to light as the child becomes older [24]. In some less severe cases the children were only followed until hospital discharge. Due to the geography and limited distribution of specialist paediatric neurology and rehabilitation services in NSW it is likely that the children would return to one of the two children’s hospitals if they required these services and would then have come to our attention during follow up. We cannot exclude relocation of the patient’s families from the catchment area of the paediatric hospitals leading them to seek rehabilitation services elsewhere.

Our study includes patients treated by a single HEMS service operating within a comprehensive EMS system. It is possible that the patients selected for HEMS dispatch are not representative of all paediatric drownings in the catchment area of the service. As it is known from the previous dispatch system study [6] that all severe paediatric drowning cases occurring prior to March 2011 were identified we are confident that the present study includes all severe drowning cases occurring during the operational hours of the service up to that date although it is possible that less severe cases were missed. Four severe drowning cases occurred after March 2011 when P-HEMS access to the CAD system was withdrawn that are not reported in this study. It is possible that these patients were different than those reported or that the different prehospital treatment provided may have produced different outcomes than that observed in this series.

## Conclusion

P-HEMS played a significant role in the management of severe paediatric drowning in this case series. Requirement for interventions that are only provided by P-HEMS in the Sydney EMS system, specifically anaesthesia facilitated intubation, were high and all cases identified for P-HEMS response were transferred directly to a paediatric specialist centre. Discontinuation of the P-HEMS direct case identification system that operated during the majority of the study period resulted in deterioration in system performance with some paediatric drowning patients not identified for P-HEMS response being transported to adult hospitals.

## Abbreviations

CPB: Cardiopulmonary Bypass
CPR: Cardiopulmonary Resuscitation
ED: Emergency Department
EMS: Emergency Medical Service
GCS: Glasgow Coma Scale
HEMS: Helicopter Emergency Medical Service
ISS: Injury Severity Score
NSW: New South Wales
PCPC: Paediatric Cerebral Performance Category
P-HEMS: Physician Staffed Helicopter Emergency Medical Service
PICU: Paediatric Intensive Care Unit
ROSC: Return of Spontaneous Circulation
